# *Toxoplasma gondii* Soluble Tachyzoite Antigen Triggers Protective Mechanisms against Fatal Intestinal Pathology in Oral Infection of C57BL/6 Mice

**DOI:** 10.1371/journal.pone.0075138

**Published:** 2013-09-24

**Authors:** Luciana Benevides, Cristina R. Cardoso, Cristiane M. Milanezi, Letícia S. Castro-Filice, Paulo V. C. Barenco, Romulo O. Sousa, Rosangela M. Rodrigues, José R. Mineo, João S. Silva, Neide M. Silva

**Affiliations:** 1 Department of Biochemistry and Immunology, School of Medicine of Ribeirão Preto, University of São Paulo, Ribeirão Preto, SP, Brazil; 2 Department of Clinical Analyses Toxicology Bromatologics, Ribeirão Preto College of Pharmaceutical Sciences, University of São Paulo, Ribeirão Preto, SP, Brazil; 3 Institute of Biomedical Sciences, Federal University of Uberlândia, Uberlândia, MG, Brazil; 4 Federal University of Goiás, Goiás, Brazil; University of California, Riverside, United States of America

## Abstract

*Toxoplasma gondii* induces a potent IL-12 response early in infection that results in IFN-γ-dependent control of parasite growth. It was previously shown that *T. gondii* soluble tachyzoite antigen (STAg) injected 48 hr before intraperitoneal infection reduces lipoxin A_4_ and 5-lipoxygenase (5-LO)-dependent systemic IL-12 and IFN-γ production as well as hepatic immunopathology. This study investigated the ability of STAg-pretreatment to control the fatal intestinal pathology that develops in C57BL/6 mice orally infected with 100 *T. gondii* cysts. STAg-pretreatment prolonged the animals’ survival by decreasing tissue parasitism and pathology, mainly in the ilea. Protection was associated with decreases in the systemic IFN-γ levels and IFN-γ and TNF message levels in the ilea and with increased TGF-β production in this tissue, but protection was independent of 5-LO and IL-4. STAg-pretreatment decreased CD4^+^ T cell, NK cell, CD11b^+^ monocyte and CD11b^+^CD11c^+^ dendritic cell numbers in the lamina propria and increased CD8^+^ T cells in the intestinal epithelial compartment. In parallel, decreases were observed in iNOS and IL-17 expression in this organ. These results demonstrate that pretreatment with STAg can induce the recruitment of protective CD8^+^ T cells to the intraepithelial compartment and decrease proinflammatory immune mechanisms that promote intestinal pathology in *T. gondii* infection.

## Introduction

C57BL/6 mice die within 13 days of peroral infection with 100 cysts of *T. gondii* strain ME-49 after developing inflammatory pathology that resembles the lesions seen in human inflammatory bowel diseases (IBD), particularly Crohn’s disease [1]. Mortality is related to an overwhelming Th1-like immune response with massive necrosis of the villi and mucosal cells in the ileum, with CD4^+^ T cells, IFN-γ, TNF and nitric oxide (NO) mediating the development of intestinal lesions [1,2]. Also, it was verified that CCR2-dependent intraepithelial lymphocytes mediate inflammatory gut pathology in oral *T. gondii* infection [3]. Activation of CD4^+^ T cells by IL-12p40 and, to a lesser extent, IL-18 was found to be required for the development of intestinal lesions following oral *T. gondii* infection, although IL-12 is dominant over IL-18 in host defence against parasite replication [4]. IL-23, which shares in common with IL-12 the p40 subunit, IL-12Rβ1, and components of signal transduction [5], is associated with Th17 responses, yet the role of IL-17 in parasite-induced ileitis is unclear; severe intestinal immunopathology was found in IL-17A^-/-^ mice following oral infection with 100 ME-49 cysts [6], whereas IL-17RA^-/-^ and IL-17R^-/-^ mice presented decreased ileitis when orally infected with 30 and 15 cysts of the 76K strain, respectively [7,8].

Within a few hours of injecting mice with live tachyzoites or soluble tachyzoite antigen (STAg), IL-12p40-producing cells, the majority of which are CD11c^+^ dendritic cells (DC), are observed in the T cell areas of the spleen; IL-12 synthesis is rapid, intense and relatively short lived, returning to baseline levels by 24 hr post-injection [9]. These DC become non-responsive to secondary administration of STAg and persist in this state for approximately 1 week [10]. The arachidonic acid metabolite lipoxin (LX) A_4_, generated by a 5-lipoxygenase (LO)-dependent pathway, is one mediator shown to be responsible for DC non-responsiveness to secondary STAg stimulation, a property that was correlated with reduced CCR5 expression [11].

In the present study, we investigated whether the STAg effect could protect against intestinal immunopathology induced by toxoplasmosis. To address this question, animals were treated with STAg and orally infected 48 hr later with 100 *T. gondii* ME-49 cysts; mortality, morbidity and immunological parameters were then monitored. STAg-pretreatment prolonged animal survival and decreased intestinal pathology through mechanisms independent of IL-4, IL-10 and 5-LO. The protective mechanisms induced by STAg were related to a decrease in CD4^+^ T cells in the lamina propria (LP) and to an increase in CD8^+^ T cells in the intestinal intraepithelial compartment.

## Materials and Methods

### Ethics statement

All animal experiments were performed in accordance to Brazilian Government’s ethical and animal experiment regulations. The experimental procedures were approved by the animal ethics committee of the São Paulo University (process 072/2006). All efforts were made to minimize animal suffering and the numbers of mice required for each experiment.

### Animals

Female 8- to 10 week-old BALB/c, C57BL/6 (WT), Swiss, Swiss athymic nude, C57BL/6 IL-4-deficient (IL-4^-/-^), IL-12p40-deficient (IL-12p40^-/-^), myeloid differentiation factor 88-deficient (MyD88^-/-^) and chemokine receptor 5-deficient (CCR5^-/-^) mice, as well as C57BL/6 MHC class II-deficient (MHC II^-/-^) mice lacking CD4^+^ T cells [12], C57BL/6 β2-microglobulin-deficient mice (MHC I^-/-^) lacking CD8^+^ T cells [13] and 5-LO-deficient mice (5-LO^-/-^) on the 129/SvEvTac background, were bred and maintained under standard conditions in the animal facility at the University of São Paulo, Brazil.

### Parasites and STAg preparation

The low-virulent ME-49 strain of *T. gondii* was used to infect animals. Cysts were harvested from the brains of C57BL/6 mice that had been inoculated 1 month previously with approximately 10 cysts by the intraperitoneal route (i.p.). To prepare STAg, RH strain tachyzoites were cultured in human foreskin fibroblasts and the parasites sonicated and centrifuged, and the supernatant was collected and prepared as previously described [14].

### Experimental procedure and histological preparation

Mice were intraperitoneally injected with 25 µg of STAg per mouse or with phosphate-buffered saline (PBS) and orally infected 48 hr later with 100 *T. gondii* cysts. The mice were analysed on day 8 post-infection (p.i.). Groups of 5 mice were injected with anesthetics Ketamine (Syntec Brasil Ltda, SP, Brazil)/Xylazine (Schering-Plough Coopers, SP, Brazil) by i.p. route and killed by cervical dislocation on day 8 post-infection (p.i.). Blood samples were collected for serological assays. Tissue samples, of brain, small intestine, lung, liver, mesenteric lymph node and spleen were collected, fixed in 10% buffered formalin, and processed routinely for paraffin embedding and sectioning. The peripheral organs and the brain were examined histologically as previously shown [15]. For each organ, tissue sections of 4 µm thickness were mounted on slides and the sections stained with haematoxylin and eosin. The histological analysis was done using a 40 x objective in a blind manner by two researchers. The inflammatory score was represented as arbitrary units: 0-2, mild; 2-4, moderate; 4-6, severe; and above 6, very severe. In experiments to detect IL-17 in the ileum, the organ was processed as described above, was embedded in Tissue Tek (OCT, Milles, -Inc, Zoeterwoude, The Netherlands), and frozen at -80°C.

### Immunohistochemical analysis for detection of tissue parasitism, iNOS or IL-17

Tissue parasitism was quantified by immunohistochemistry as previously described [15]. Deparaffinized sections were incubated overnight at 4°C with polyclonal rabbit antibody against antigens of *T. gondii* ME-49 strain or non-immune rabbit serum as control, and then with biotinylated goat anti-rabbit antibodies (Sigma Aldrich, St. Louis, USA). To detect iNOS, deparaffinised sections were incubated first with rabbit anti-iNOS antibody (Santa Cruz Biotechnology, Santa Cruz, CA, USA) and then with biotin-labelled goat anti-rabbit antibody (Sigma Aldrich, St. Louis, MO, USA). For IL-17 detection, sections were incubated first with goat anti-IL-17 (Santa Cruz) and then with biotin-labelled donkey anti-goat antibody (Santa Cruz). Next, all sections were incubated with avidin-biotin-peroxidase complex (ABC kit, PK-4000), and the colour developed with 3,3'-diaminobenzidine (Sigma). The tissue parasitism was scored by counting the number of parasitophorous vacuoles per section in the small intestine and brain or from forty microscopic fields in the lung, liver, mesenteric lymph node and spleen using a 40 x objective. The analyses were done in two histological sections of each mouse from at least three mice per group.

### Cytokine measurement in serum samples and intestinal homogenates

Cytokine concentrations were measured by ELISA using assay kits for IL-12p70, IFN-γ, IL-10, TGF-β, TNF (OpTEIA, BD Bioscience, San Diego, CA, USA) and IL-4 (Duoset R&D Systems, Minneapolis, MN, USA) according to the manufacturer’s instructions. The ileum tissue samples (100 mg) obtained from C57BL/6 mice were homogenized with an Omni TH homogenizer in 0.5 ml of PBS containing protease inhibitors (0.1 mM phenylmethylsulfonyl fluoride, 0.1 mM benzamidine chloride, 10 µg/ml of aprotinin A and 100 µg/ml of leupeptin). Each sample was centrifuged for 10 min at 3000 x g, and the supernatant used for ELISA. The cytokine concentrations in the samples were calculated based on standard curve generated with murine recombinant cytokines. The sensitivity of detection in the ELISAs was 31.25 pg/ml for IL-12p70, 15.63 pg/ml for IFN-γ, TNF and IL-10, 62.5 pg/ml for TGF-β, and 3.9 pg/ml for IL-4.

### Flow cytometry assay

The small intestine inflammatory infiltrates were evaluated as intraepithelial lymphocytes (IEL) and lamina propria leucocytes (LPL), as described [16]. Viability was assessed by Trypan blue exclusion and cells were counted in a Neubauer chamber. Leucocytes obtained were incubated with anti-CD16/CD32 mAb (Fc block, Clone 2.4G2-Pharmingen, San Jose, CA, USA), followed by incubation with the following antibodies: anti-CD8, anti-CD19, anti-CD11c, anti-CD25 FITC-conjugated (BD Biosciences Pharmingen); anti-Pan NK, anti-CD4, anti-TCRγδ, anti-CD11b (Southern Biotechnologies Associates Inc., Birmingham, Al, USA), anti-CD8 (BD) PE conjugated and anti-CD3 PerCP-labelled antibodies (BD). Leucocytes were acquired (FACScanTM and CELLQuestTM software; BD) according to size and granularity scatter dot plot. The results showed the mean ± SD of the number of cells obtained from each tissue after analysis of the percentage of each antibody specific stained subpopulation within the gated cells, obtained from small gut of five different animals per group.

### T cell isolation

LP cells and IELs were isolated of the small intestine and (CD3^+^CD4^+^) and (CD3^+^CD8^+^) T cells were sorted using a FACSAria III fluorescent cell sorter (BD Biosciences, San Jose, CA). Purity of sorted cells preparations were >98%. After the sorting, the mRNA was extracted for evaluation of the IFN-γ, TGF-β and the transcription factor RORγt, the master regulator of Th17 cells [17] gene expression, as described below.

### In vitro restimulation and intracellular cytokine detection cells

Isolated cells from LP or intraepithelial compartment were cultured in RPMI 1640 supplemented with 5% FBS, penicillin/ streptomycin (Gibco - Invitrogen), HEPES (Sigma Aldrich), L-glutamine (Sigma Aldrich), nonessential amino acids (Gibco - Invitrogen), and 50 mM of β-mercaptoethanol (Merck) in the presence of 500 ng/mL Ionomicin (Sigma Aldrich), 50 ng/mL phorbol 12-myristrate 13 acetate (PMA - Sigma Aldrich) and 1 µg/mL Brefeldin (Golgi Stop^TM^ – BD – Biociences). After 2 hr, cells were harvested, labeled for surface molecules CD3 (145-2C11), CD4 (RM4-5) and CD8 (clone 53-6.7) (BD – Biosciences), and cell membranes were permeabilized with Cytofix/Cytoperm™ Fixation/Permeabilization Solution Kit (BD – Biosciences) according to the manufacture’s instruction. Intracellular cytokines were stained with fluorochrome-conjugated antibodies against IFN-γ (XMG1.2) (BD – Biosciences) and IL-17 (TC11-18H10) (BD – Biosciences).

### Real-time polymerase chain reaction

Fragments containing 100 mg were obtained from ilea and the RNA was extracted using RNA extraction kit (Promega, Madison, WI, USA), and complementary DNA (cDNA) was synthesized using 1 µg of RNA through a reverse transcription reaction (M-MLV reverse transcriptase, Promega). Real-PCR quantitative mRNA analyses were performed on the ABI Prism 7500 Sequence Detection System using SYBR green fluorescence (Applied Biosystems, Warrington, UK). The primers were the following: β-actin 5´-AGCTGCGTTTTACACCCTTT-3´, 5´-AAGCCATGCCAATGTTGTCT-3´; TNF 5´-TGTGCTCAGAGCTTTCAACAA-3´, 5´-CTTGATGGTGGTGCATGAGA-3´; IL-12p40 5´-AGCACCAGCTTCTTCATCAGG-3´, 5´-GCGCTGGATTCGAACAAAG-3´; IFN-γ 5´-GCATCTTGGCTTTGCAGCT-3´, 5´-CCTTTTTCGCCTTGCTGTTG-3´; IL-10 5´-TGGACAACATACTGCTAACC-3´, 5´-GGATCATTTCCGATAAGGCT-3´; RORγt 5´-TGGAAGATGTGGACTTCGTTT -3´, 5´- TGGTTCCCCAAGTTCAGGAT -3´; TGF-β 5´-TGAACCAAGGAGACGGAATACA -3´, 5´-GGAGTTTGTTATCTTTGC TGTCACA-3´. SYBR Green PCR Master Mix (Applied Biosystems), 0.1-0.2 µg/µL specific primers, and 2.5 ng of cDNA were used in each reaction. Threshold for positivity was determined based on negative controls. The results were demonstrated as mRNA expression, relative to non-infected mice. Calculations to determine the relative level of gene expression were made according to the instructions from Applied Biosystems User’s Bulletin 2 (P/N 4303859), by reference to the β-actin in each sample, using the threshold cycle (C_t_) method.

### Statistical analysis

The Kaplan-Meier method was used to compare the survival rates of experimental groups and the survival curves were compared using logrank and chi square tests. The comparisons between PBS- or STAg-pretreatment were analyzed by Student’s t or Mann-Whitney test, whereas comparisons between non-infected, PBS- or STAg-pretreated mice were analyzed by ANOVA (one-way) and Bonferroni multiple comparison post-tests or Kruskal Wallis tests, when appropriate. Values of *P* < 0.05 were considered statistically significant.

## Results

### Previous STAg treatment protects against oral *T. gondii* infection

**Figure 1:**
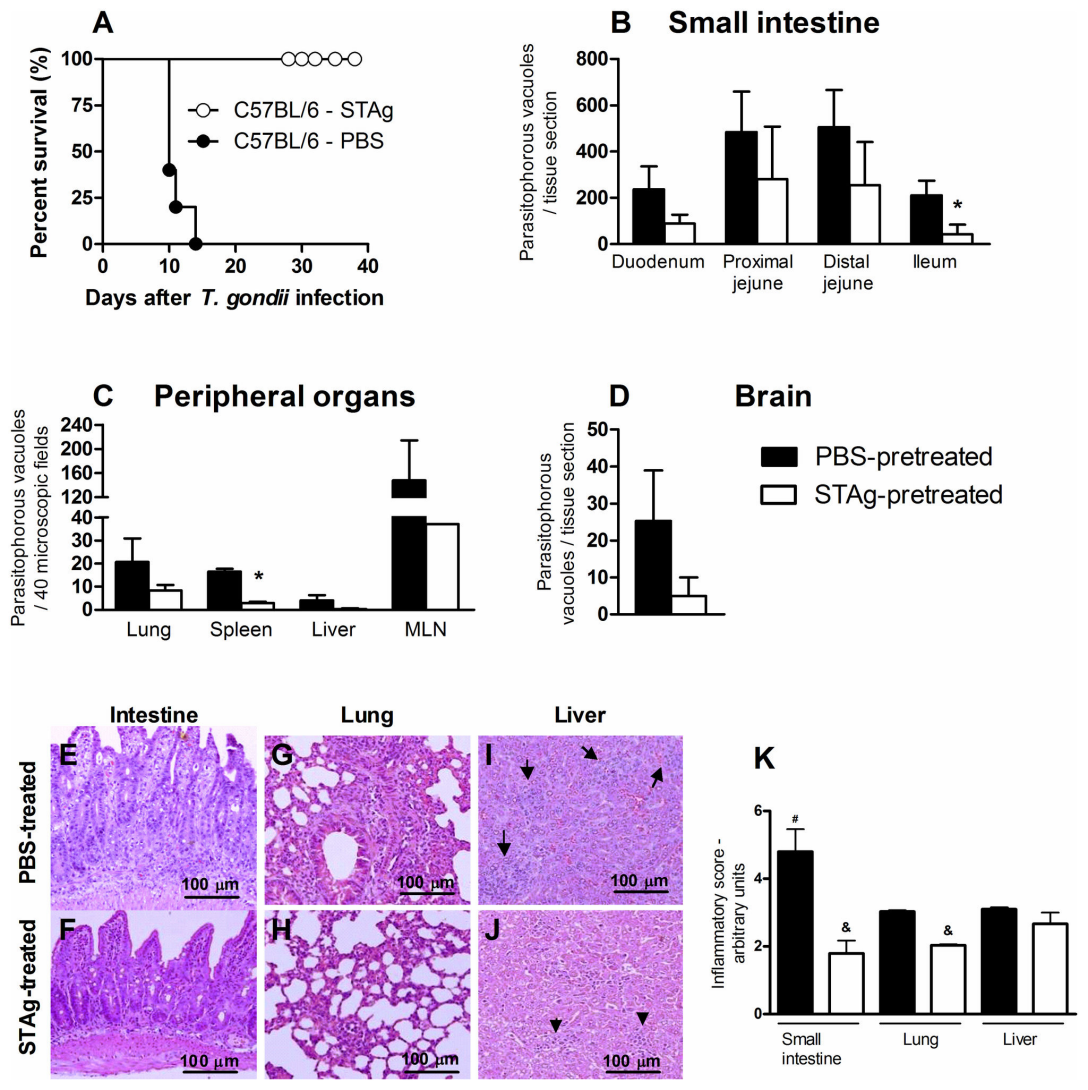
Mortality rates, tissue parasitism and inflammatory changes of STAg-pretreated C57BL/6 mice orally *T. gondii* infected. The mortality rate for 8 mice from each group was determined (A). STAg-pretreated mice were significantly more resistant to toxoplasmosis than PBS-pretreated mice (χ^2^=10.03; *P* = .0015; df = 1). Tissue parasitism in the small intestine (B), peripheral organs (C) and brain (D) were detected on day 8 p.i. by immunohistochemistry staining and scored by counting the number of parasitophorous vacuoles per tissue section in the small intestine and brain and per 40 microscopic fields in the other peripheral organs, using a 40 x objective. The small intestine (E,F), lung (G,H) and liver (I,J) of PBS- and STAg-pretreated mice were stained by H&E and analyzed for histological changes. The inflammatory foci in the liver are shown (arrows). Bar scale, 100 µm. The data of inflammatory scores in the organs were obtained by analyzing 40 microscopic fields per section on six sections using a 40 x objective from each mouse (K). Data are representative of at least two independent experiments of 5 mice per group that provided similar results. *p < 0.05 (Significantly different from values obtained from PBS-pretreated mice, Unpaired Student’s *t*-test). ^&^p < 0.03 (Significantly different from values obtained from PBS-pretreated mice, Mann Whitney test); ^#^ *p* < 0.05 (Significantly different from values obtained from lung and liver of PBS-pretreated mice, Kruskal-Wallis test and Dunn’s multiple comparisons post-test).

To investigate the role of the previous STAg treatment in oral *T. gondii* infection, the animals were treated with STAg and 48 hr later, inoculated with 100 cysts of the ME-49 strain. Infected C57BL/6 mice pretreated with PBS were highly susceptible to infection. STAg-pretreated mice, in contrast, became resistant, and all survived for at least 40 days p.i. (Figure 1A). To determine whether the STAg-induced protection was related to decreased tissue parasitism, we measured the parasite load in the organs by immunohistochemistry assays. Notably, STAg-pretreatment decreased the parasitism in the small intestine, mainly in the ileum, and in the spleen (Figure 1B and 1C). Although not statistically significant, decreases in parasitism in the lung, liver, mesenteric lymph node and brain were seen with STAg-pretreatment (Figure 1C and 1D). With regard to inflammatory changes, PBS-pretreated mice had greater inflammation in the small intestine, particularly the ileum, than in the other organs at day 8 p.i. (Figure 1K) and exhibited intense infiltration of inflammatory cells into the LP, epithelium and submucosa (Figure 1E). In contrast, the small intestines of STAg-pretreated mice presented only mild inflammatory changes (Figure 1F). The lung of PBS-pretreated mice showed large inflammatory infiltrates in the alveolar walls (Figure 1G), whereas STAg-pretreatment was able to decrease the inflammatory changes in the lung (Figure 1H and 1K) and in the liver, although the latter effect was not statistically significant (Figure 1I-1J and 1K). These findings suggest that STAg-pretreatment can, at least in part, control tissue parasitism, ameliorate inflammatory changes and improve parasite resistance in *T. gondii*-infected mice.

To determine whether the protective effect of STAg was parasite specific, BALB/c mice were pretreated with STAg and infected with *Trypanosoma cruzi*. In this experiment, STAg- and PBS-pretreated mice presented the same pattern of susceptibility (Figure S1A). In fact, STAg-pretreated mice presented higher parasitemia compared with PBS-pretreated mice on days 11 and 13 p.i. (Figure S1B). Thus, the protective mechanism induced by STAg is *T. gondii* specific.

### STAg decreases IFN-γ in the serum and modulates the IFN-γ gene expression and production and increases TGF-β in the ileum

**Figure 2:**
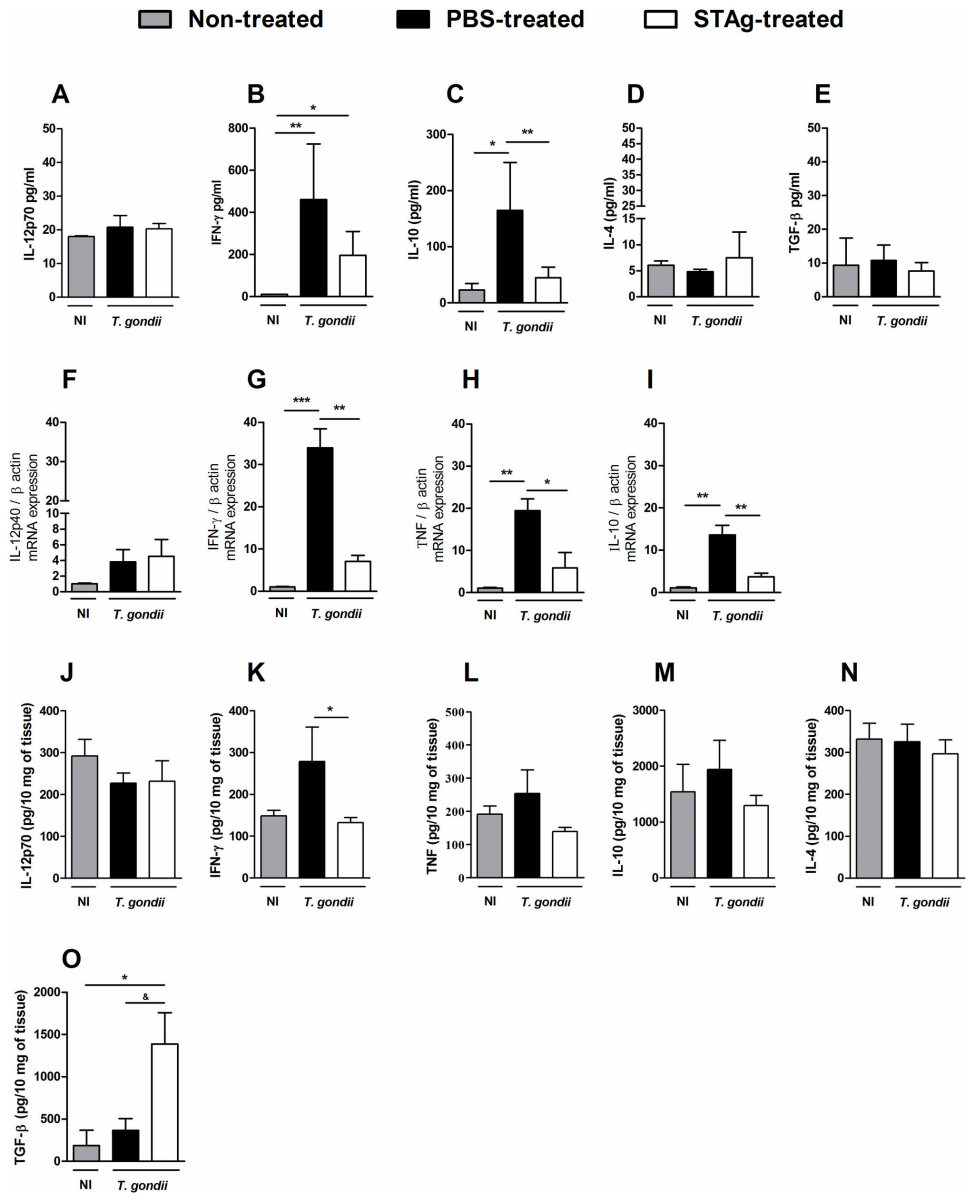
Cytokine levels in the sera and mRNA expression and cytokine production in the ilea of STAg-pretreated orally *T. gondii* infected C57BL/6 mice. The levels of IL-12p70 (A), IFN-γ (B), IL-10 (C), IL-4 (D) and TGF-β (E) in serum samples, and IL-12p70 (J), IFN-γ (K), TNF (L), IL-10 (M), IL-4 (N) and TGF-β (O) in the ilea were measured on day 8 p.i. by ELISA. The values of cytokine production were the mean and SD of 5 mice per data point. The experiments were repeated twice and provided similar results. The cytokine IL-12p40 (F), IFN-γ (G), TNF (H) and IL-10 (I) transcript expressions in the ilea of STAg-pretreated and infected mice was quantified by qPCR on day 8 p.i. Results are demonstrated as mRNA expression of PBS- or STAg-pretreated and *T. gondii*-infected mice, relative to non-infected mice. The relative levels of gene expression were calculated by reference to the β-actin in each sample, using the threshold cycle (C_t_) method; and the data (mean ± SD) represent values from one experiment representative of three independent experiments. * *p* < 0.05, **p < 0.01, ***p < 0.001 (ANOVA and Bonferroni multiple comparisons post-test). ^&^ *p* < 0.05 (Significantly different from values obtained from PBS-pretreated mice, Unpaired Student’s *t*-test). NI, non-infected.

As it was previously shown that STAg-pretreatment decreased the systemic IFN-γ levels in intraperitoneal *T. gondii*-infection [10] and TGF-β have shown an important role in the protection against the inflammation induced by *T. gondii* in the small intestine [18,19], the systemic and local levels of cytokines were measured. Similar systemic IL-12p70 levels were found in infected mice pretreated with PBS or STAg (Figure 2A) on day 8 p.i. *T. gondii* infection induced a significant increase in serum IFN-γ levels in both PBS- and STAg-pretreated mice; however, STAg-pretreatment resulted in lower IFN-γ levels compared to PBS-pretreatment (Figure 2B). IL-10 levels also increased with *T. gondii* infection in both PBS- and STAg-pretreated mice, but again, IL-10 levels were lower in STAg-pretreated mice versus PBS-pretreated mice (Figure 2C). The amounts of IL-4 (Figure 2D) or TGF-β (Figure 2E) detected in the serum on day 8 p.i. were not altered by infection.

In the ileum it was measured the cytokine mRNA expression by qPCR and protein production by ELISA. It was verified similar IL-12p40 mRNA expression in PBS- and STAg-pretreated mice on day 8 p.i. (Figure 2F). PBS-pretreated infected C57BL/6 mice had higher levels of IFN-γ and TNF messages in the ileum compared with non-infected and with STAg-pretreated infected mice (Figure 2G-2H). Although not a statistically significant change, the STAg-pretreated mice also had higher IFN-γ and TNF message levels compared to non-infected mice. Increased IL-10 message levels in the ileum were also observed on day 8 of *T. gondii* infection, and the increase was statistically significant in the PBS-pretreated group (Figure 2I). However, the levels of IL-10 message in STAg-pretreated infected mice were significantly lower compared to levels in PBS-pretreated infected mice (Figure 2I).

Related to cytokine production and in accordance with IL-12p40 mRNA expression, similar levels of IL-12p70 was observed in the ileum of PBS- or STAg-pretreated and infected mice (Figure 2J); and lower IFN-γ levels were detected with STAg-pretreament (Figure 2K). Additionally, no statistical difference was observed in TNF, IL-10 and IL-4 production in the organ under infection and STAg-pretreatment (Figure 2L, 2M and 2N), despite TNF detection presented a trend to be lower in STAg-pretreated compared to PBS-pretreated mice (Figure 2L). Interestingly, the levels of TGF-β increased in the ilea of infected mice (Figure 2O). Furthermore, TGF-β levels were higher in STAg-pretreated infected mice compared to non-infected or PBS-pretreated infected mice (Figure 2O). Together these data indicate that the lower inflammation in STAg-pretread mice could be related to lower systemic production and local intestinal mRNA expression and production of IFN-γ and higher TGF-β production.

### STAg-pretreatment decreases iNOS and IL-17 expression in the ileum during *T. gondii* infection

**Figure 3:**
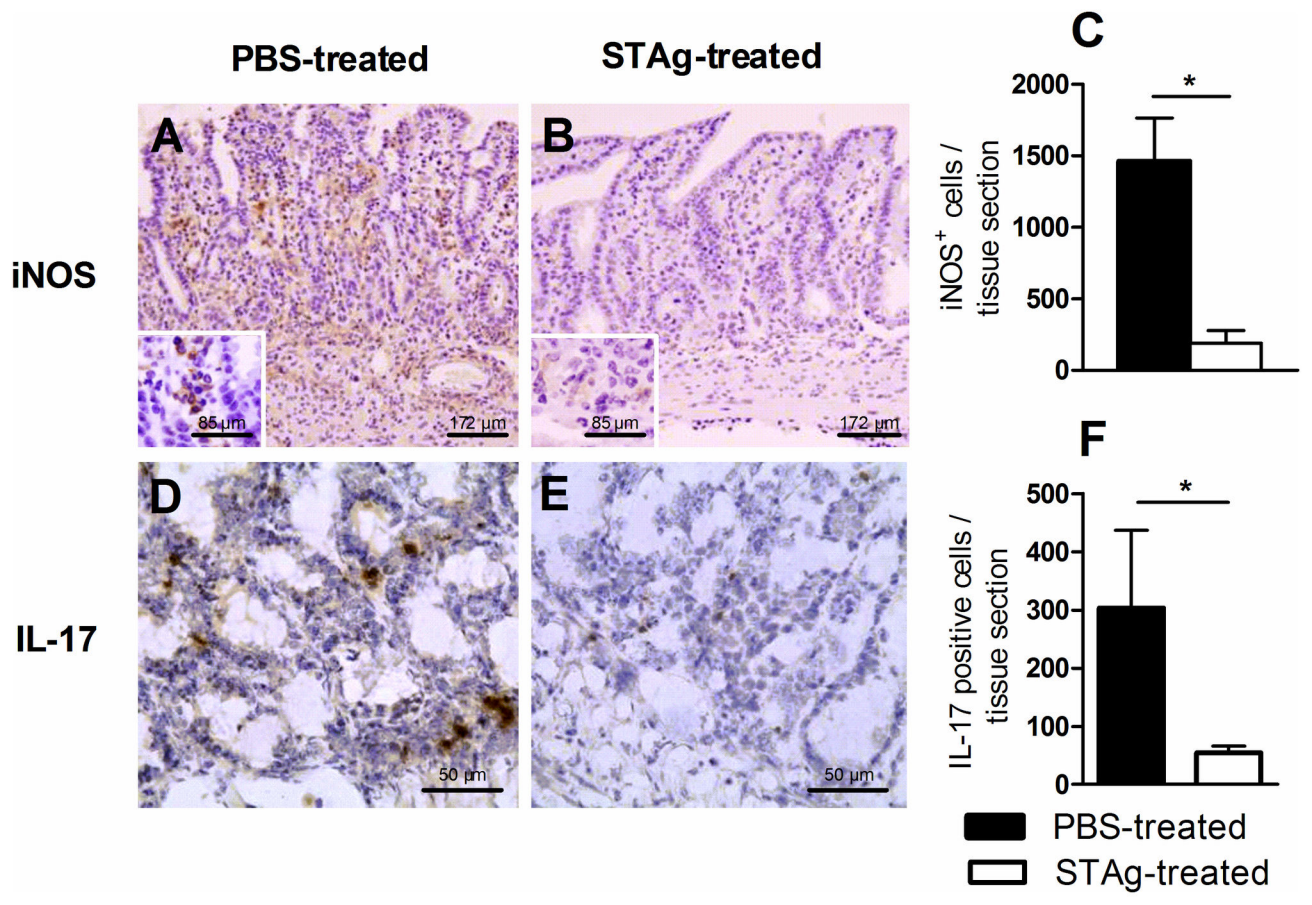
Immunohistochemical photomicrographs and quantification of iNOS and IL-17 positive cells of STAg-pretreated and infected mice. The C57BL/6 mice were STAg pretreated and infected and the ilea were collected on day 8 p.i. for iNOS or IL-17 immunostaining. The quantification of iNOS+ (A, B and C) or IL-17+ (D, E and F) cells was performed in 40 microscopic fields per tissue section using a 40 x objective. *p < 0.05 (Statistically different from values observed for the same cell phenotype obtained from PBS-pretreated mice, Unpaired Student’s *t*-test).

TNF-α, nitric oxide and IFN-γ are all critical for development of necrosis in the small intestine and early mortality in C57BL/6 mice orally infected with high parasite load of ME-49 strain of *T. gondii* [2], and IL-17 is involved in ileitis in mice infected with cysts of the 76K strain by oral route [7,8]. To assess whether the smaller inflammatory response observed in the ileum of STAg-pretreated infected mice was related to down-regulation of iNOS or IL-17, we measured the number of cells expressing these inflammatory mediators. STAg-pretreatment was found to decrease the numbers of cells expressing iNOS or IL-17 in the ileum on day 8 p.i. compared to PBS-pretreated animals (Figure 3A-3F). Thus, the lower numbers of cells expressing iNOS and/or IL-17 could be contributing to the smaller intestinal inflammation in STAg-pretreated mice.

### Mechanisms of STAg-induced protection are independent of 5-LO and IL-4

**Figure 4:**
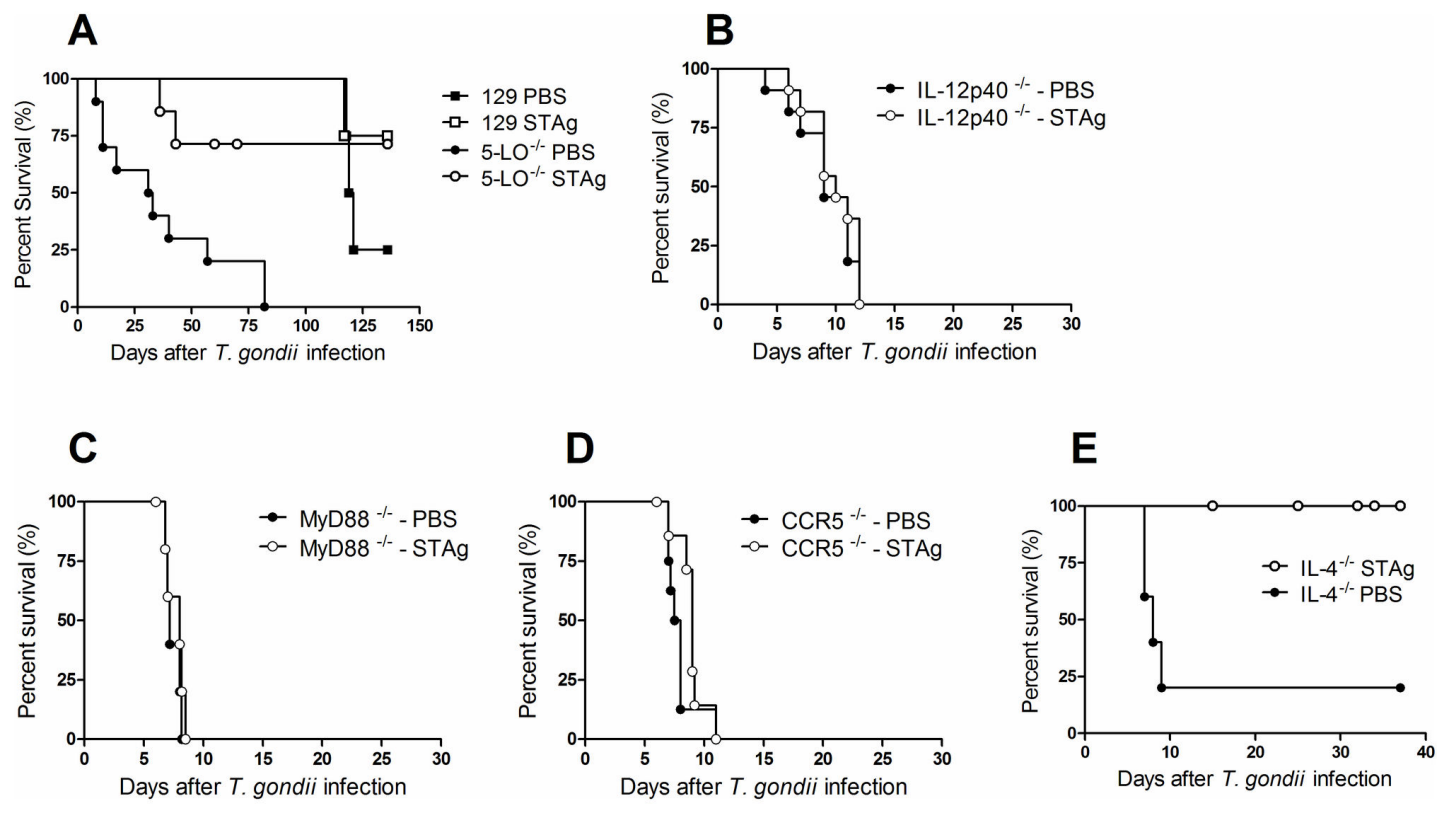
Mortality rates of 5 LO^-/-^, IL-4^-/-^, IL-12p40^-/-^, MyD88^-/-^ and CCR5^-/-^ STAg-pretreated and infected mice. 5 LO^-/-^ (A), IL-12p40^-/-^ (B), MyD88^-/-^ (C), CCR5^-/-^ (D) and IL-4^-/-^ (E) mice were pretreated with STAg and 48h later were infected with 100 *T. gondii* cysts by oral route. The C57BL/6 that were the WT mice of IL-4^-/-^, IL-12p40^-/-^, MyD88^-/-^ and CCR5^-/-^ and 129/SvEvTac that were the WT of 5 LO^-/-^ mice were also examined. The mortality rate for 8 mice from each group was determined. 5-LO^-/-^ (A), or IL-4^-/-^ (E) STAg-pretreated were significantly more resistant to toxoplasmosis than PBS-pretreated mice (χ^2^=9.171; *p* = 0.0018; df = 1; related to 5-LO^-/-^ mice; and χ^2^=6.198; *p* = 0.0128; df = 1; related to IL-4^-/-^ mice).

We examined the effects of STAg-pretreatment in mice deficient for 5-LO, IL-12p40, MyD88 and CCR5, based on evidence that these factors are involved in IL-12 production during *T. gondii* infection and that IL-12 has an important role in the development of parasite intestinal immunopathology [4,20,21]. We observed that STAg-pretreated 5-LO^-/-^ mice were resistant to infection compared with PBS-pretreated 5-LO^-/-^ mice (Figure 4A). As IL-12 and MyD88 are crucial to protection against parasitic infection, IL-12p40^-/-^, MyD88^-/-^ and CCR5^-/-^ mice were unsurprisingly highly susceptible to *T. gondii* infection (Figure 4B-4D).

The effect of STAg-pretreatment on the mortality of IL-4^-/-^ mice was also examined. We observed that STAg-pretreated IL-4^-/-^ mice efficiently controlled the infection (Figure 4E). These results suggest that STAg-dependent protection against oral *T. gondii*-infection involves mechanisms that are independent of 5-LO and IL-4.

### STAg-pretreatment decreases CD4^+^ T cells with proinflammatory profile in the LP and increases intraepithelial CD8^+^ T cell infiltration of the small intestine

**Figure 5:**
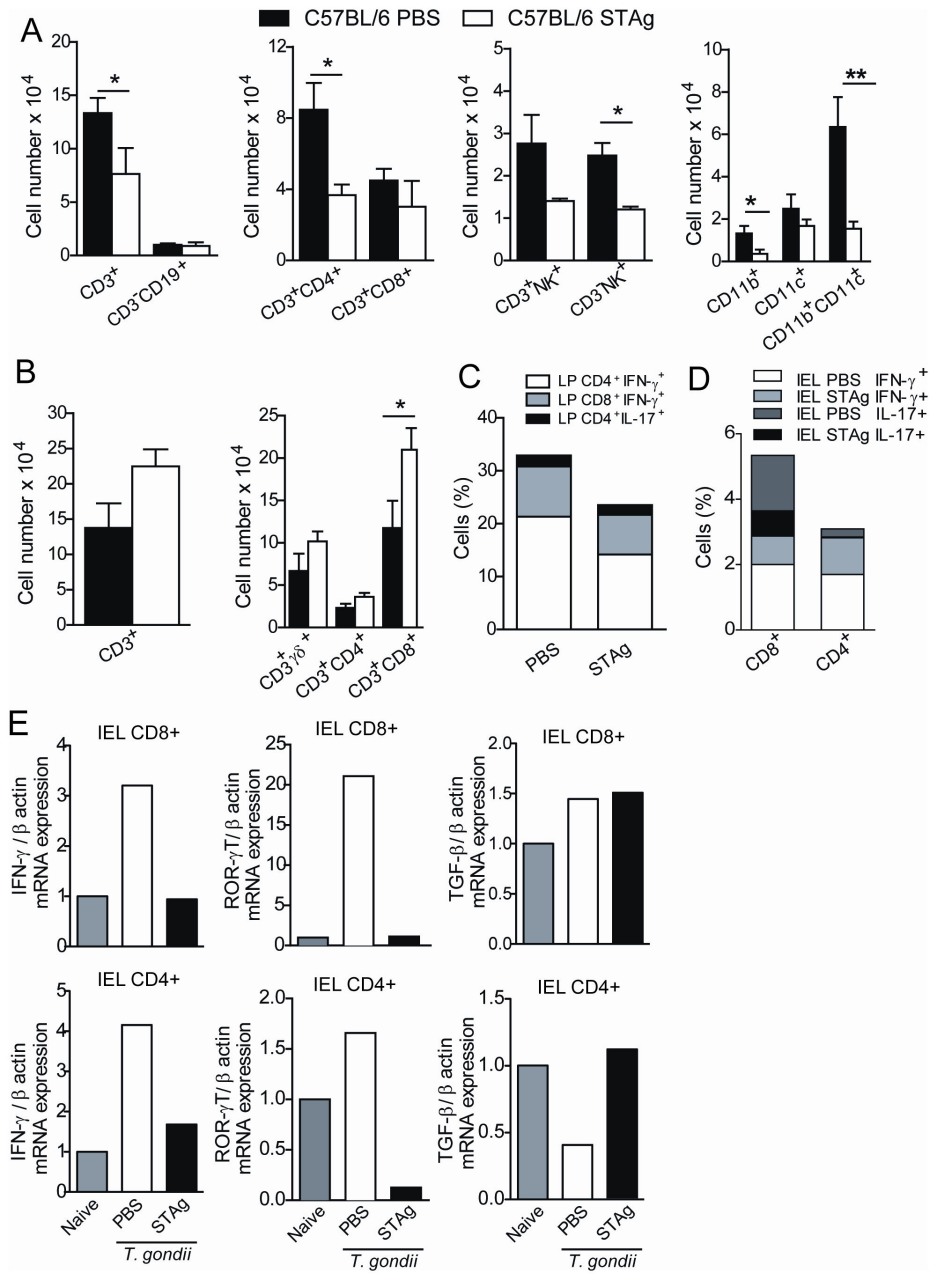
Phenotypic characterization of leukocytes in the small intestine of STAg-pretreated and infected C57BL/6 mice. The leukocytes were obtained from the small intestine on day 8 p.i. as described on 'Material and methods' section. The number (mean ± SD) of CD3^+^, CD3^+^CD4^+^, CD3^+^CD8^+^, CD19^+^, CD3^+^NK^+^, NK^+^ and CD11b^+^, CD11c^+^, CD11b^+^CD11c^+^ (A) leukocytes were analyzed in the LP. The numbers of CD3^+^, CD3^+^γδ^+^, CD3^+^CD4^+^, CD3^+^CD8^+^ (B) lymphocytes were analyzed in the intraepithelial compartment. The intracellular IFN-γ and IL-17 production, after *in* *vitro* stimulation with PMA/Ionomicin were detected in cells from the LP (C) and intraepithelial compartment (D) of the small intestine from PBS- or STAg-pretreated C57BL/6 mice on day 8 p.i. The IFN-γ, TGF-β, and RORγt (E) transcript expressions in CD4^+^ or CD8^+^ IELs from a pool obtained of 4 mice per group of naïve, PBS- or STAg-pretreated and infected mice was quantified by qPCR on day 8 p.i. Data are representative of at least two independent experiments of 5 mice per group. *p < 0.05, **p < 0.01 (Statistically different from values observed for the same cell phenotype obtained from PBS-pretreated mice, Unpaired Student’s *t*-test).

As it was previously shown that CD8^+^ intestinal intraepithelial lymphocytes prevent *T. gondii*-driven inflammation of LP CD4^+^ T cells [19], C57BL/6 mice were STAg-treated and infected and the leukocyte subpopulation were analyzed in the lamina propria and intraepithelial compartment. In addition, in order to verify which proinflammatory cytokine the CD3^+^CD4^+^ and CD3^+^CD8^+^ LP, and CD3^+^CD4^+^ and CD3^+^CD8^+^ IELs were producing, we detected the intracellular IFN-γ and IL-17 cytokine production by cells cultured *in vitro* collected from the small intestine of PBS or STAg-pretreated mice, and also analyzed IFN-γ, TGF-β and RORγt mRNA expression by cells from the intraepithelial compartment. *T. gondii* infection increased leukocyte numbers in the small intestine compared with non-infected mice (data not shown). Reduced migration of CD3^+^ and CD3^+^CD4^+^ T cells to the LP was observed in STAg-pretreated mice compared to the PBS-pretreated controls (Figure 5A). Additionally, smaller numbers of NK, CD11b^+^ and CD11b^+^CD11c^+^ cells were found in the LP of STAg-pretreated mice compared to PBS-pretreated mice (Figure 5A). In contrast, an analysis of IEL showed increased CD3^+^ cell migration in STAg- versus PBS-pretreated mice (Figure 5B). In STAg-pretreated mice, the intraepithelial compartment showed significantly greater numbers of CD3^+^CD8^+^ IELs compared to animals pretreated with PBS (Figure 5B). The frequency of CD3^+^CD4^+^IFNγ^+^ and CD3^+^CD8^+^ IFNγ^+^ T cells was decreased in STAg-pretreated mice in comparison with PBS-pretreated mice and, also CD3^+^CD4^+^IL-17^+^ cells was slightly decreased in the LP (Figure 5C).

The frequency of CD3^+^CD8^+^IFNγ^+^ and CD3^+^CD8^+^IL-17^+^ IELs was decreased in STAg-pretreated mice compared to PBS-pretreated mice (Figure 5D); and the CD3^+^CD4^+^IFNγ^+^ and CD3^+^CD4^+^IL-17^+^ IELs, despite in smaller numbers in comparison with CD3^+^CD8^+^ cells were also decreased in STAg-pretreated mice (Figure 5D). In accordance, it was observed smaller IFNγ and RORγt mRNA expressions in CD3^+^CD8^+^ and CD3^+^CD4^+^ IELs from animals STAg-pretreated compared to PBS-pretreated mice (Figure 5E). Interestingly, CD3^+^CD8^+^ IELs of PBS- or STAg-pretreated animals expressed TGF-β mRNA and CD3^+^CD4^+^ IELs of PBS-pretreated decreased TGF-β mRNA and STAg-pretreatment was able to maintain the cytokine expression (Figure 5E).

### CD4^+^ and CD8^+^ T cells are involved in the pathology induced by oral *T. gondii* infection

**Figure 6:**
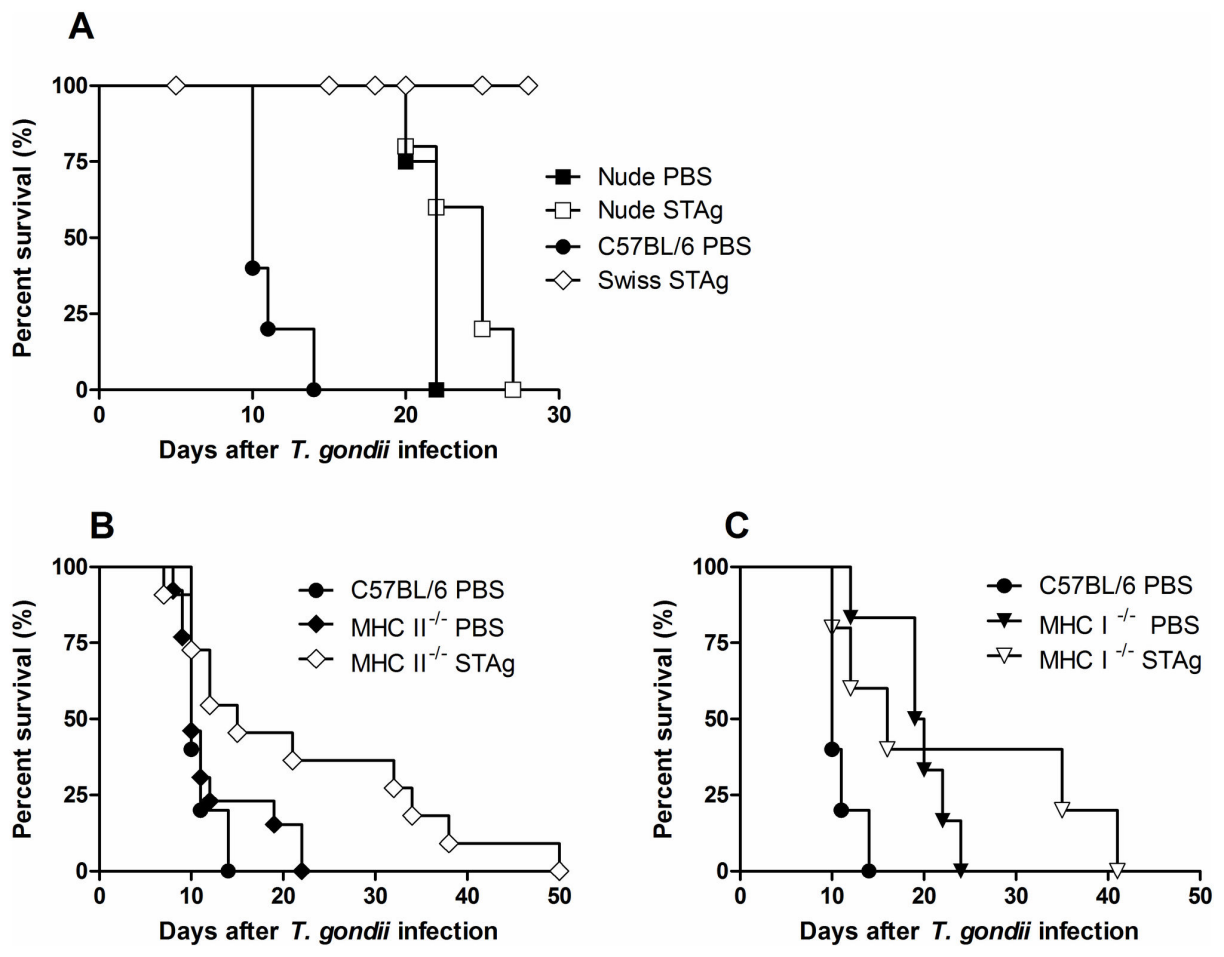
Mortality rates of nude, MHC II^-/-^ and MHC I^-/-^ STAg-pretreated and infected mice. The mortality rate for 8 mice from each group, nude (A), MHC II^-/-^ (B) and MHC I^-/-^ (C) was determined. Nude mice STAg- or PBS-pretreated survived significantly longer than euthymic control *T. gondii*-infected mice (χ^2^=10.03; *p* = 0.0015; df = 2). MHC II^-/-^ -STAg-pretreated mice were more resistant to infection than PBS-pretreated-MHC II^-/-^ or -C57BL/6 mice (χ^2^=4.493; *p* = 0.034; and χ^2^=4.120; *p* = 0.424; df = 2, respectively). MHC I^-/-^ pretreated with PBS or STAg were more resistant to infection than PBS-pretreated-C57BL/6 mice (χ^2^=9.069; *p* = 0.0026; χ^2^=3.974; *p* = 0.0462; df = 2, respectively).

Based on the observation that pretreatment of mice with STAg increases CD8^+^ T cell infiltration of the intraepithelial compartment and decreases CD4^+^ T cell infiltration of the LP, we analysed the effect of STAg on oral infection of mice lacking these cell populations. First, nude mice were pretreated with STAg and orally infected with 100 cysts. STAg- or PBS-pretreated infected nude mice survived significantly longer than infected C57BL/6 mice, although mice of both strains died in the late acute phase of infection (Figure 6A) and no difference in mortality was observed between STAg- or PBS-pretreated nude mice (Figure 6A). These results indicate that the presence of T cells predisposes mice to early death in an infection with a high parasite load; however, these cells are crucial for controlling mortality in the late acute phase.

To determine whether CD4^+^ or CD8^+^ T cells contribute to protection or promote mortality, MHC II^-/-^ or MHC I^-/-^ mice were pretreated with STAg and then infected. We observed that PBS-pretreated MHC II^-/-^ mice had similar susceptibility to infection as PBS-pretreated C57BL/6 mice, while STAg-pretreated MHC II^-/-^ mice were more resistant to infection than PBS-pretreated-MHC II^-/-^ or C57BL/6 mice (Figure 6B). PBS- or STAg-pretreated infected MHC I^-/-^ mice had higher resistance to infection than PBS-pretreated-C57BL/6 mice (Figure 6C). These data show the detrimental effect of CD8^+^ T cells on the survival of mice orally infected with a high *T. gondii* inoculum. However, both T cell types are involved in protection during the late acute and chronic phases of infection, as all animals died by day 50 p.i.

## Discussion

In the present study, we treated C57BL/6 mice with STAg and analysed its protective mechanisms when animals were infected 48 hr later with 100 cysts by oral route. We observed that when mice were STAg-pretreated and infected, they became resistant to infection. The resistance of STAg-pretreated mice was associated with reduced tissue parasitism and inflammation of the organs, particularly for the ileum. It was previously shown that lower parasite load induce less inflammation in the small intestine in oral *T. gondii* infection, such as 10 cysts by oral route induced less inflammation than 60 cysts [22]. However, so low doses of Toxoplasma, as 4 or one single-tissue cyst also induce pathology and inflammatory cytokines production [23-25]. In our investigation, in addition to the beneficial effect of the reduced parasitism, other mechanisms induced by STAg are involved in the control of the exacerbated immune response. In this context, STAg-pretreatment decreased systemic IFN-γ levels in infected mice compared to PBS-pretreated infected mice. These data are in accordance with previous experiments showing that STAg-pretreatment decreased the systemic IFN-γ levels on day 5 p.i. [10]. *T. gondii* infection induced high IL-10 levels; however, STAg-pretreatment decreased IL-10 levels relative to PBS-pretreatment, suggesting that the protective mechanism functions independently of IL-10. It has been reported that IL-4 counter-regulates type-1 immunity [26] and that TGF-β has the ability to initiate and maintain immune tolerance [27]. We verified that STAg-pretreatment did not alter systemic IL-4 or TGF-β levels in infected mice. These results suggest that the protective effect seen in STAg-pretreated mice could be related to decrease IFN-γ and occurs independently of systemic IL-10, IL-4 and TGF-β.

IL-12p40 message levels detected in the ileum on day 8 p.i. were higher compared to levels in non-infected mice, although not to a statistically significant degree. Furthermore, the observed IFN-γ, TNF and IL-10 message levels following STAg-pretreatment were significantly lower than in PBS-pretreated mice, despite being increased compared to non-infected mice. IL-10 is a cytokine usually synthesised together with IL-12 in the acute phase of *T. gondii*-infection [28], and the requirement for IL-10 in preventing immunopathology is well documented [29,30]. Despite, higher IL-12p40 and IL-10 message levels observed on day 8 of *T. gondii* infection, it was not observed a significant change in IL-12p70 or IL-10 protein levels in the ileum, indicating that the STAg-mediated protective mechanism was independent of IL-12p70 or IL-10 alterations in this time of infection. Thus, the decreased IFN-γ and TNF expression observed with STAg-pretreatment resulted in decreased gut inflammation and increased survival of infected mice, since oral infection of C57BL/6 mice with high doses of *T. gondii* induces an overwhelming intestinal Th1 response [1,31]. As TGF-β was previously shown to be produced by IEL [18,32], we measured TGF-β levels in the ileum. Pretreatment with STAg induced higher ileum TGF-β levels compared to PBS-pretreatment. TGF-β affects T cell proliferation, differentiation and survival [33,34]. Thus, TGF-β induced by STAg could decrease the viability of detrimental T cells in the small intestine, thereby contributing to decreased intestinal lesions.

Previous studies demonstrated that STAg stimulates IL-12 production by DC through binding to CCR5 [35], and the cytokine is critical to inducing IFN-γ production and conferring protection against *T. gondii* [36,37]. Conversely, IL-12 contributes to gut Th1-type immunopathology [4]. We therefore analysed disease in IL-12p40^-/-^, CCR5^-/-^ or MyD88^-/-^ mice pretreated with STAg and orally infected. Each of the STAg-pretreated infected mutant strains was highly susceptible to infection. These results demonstrated that when IL-12 signalling is absent or present at very low levels, the STAg-induced mechanism cannot protect mice against oral infection.

IL-4, a cytokine that classically counter-regulates the type-1 response, plays both protective and detrimental roles in *T. gondii* infection [38,39]. We observed that PBS-pretreated IL-4^-/-^ mice were susceptible to infection, while STAg-pretreated IL-4^-/-^ mice showed resistance. Mice deficient for 5-LO are more susceptible to infection than WT mice and develop augmented proinflammatory cytokine production and tissue pathology in intraperitoneal *T. gondii* infection [40]. The present investigation found that control of oral infection developed normally in STAg-pretreated 5-LO^-/-^ mice. Thus, STAg induces other mechanisms independent of IL-4 and 5-LO that control *T. gondii*.

It was previously shown that CD4^+^ T cells in the LP are responsible for the severe histopathological changes in the intestine that lead to tissue necrosis and subsequent mortality on day 7 of oral *T. gondii* infection [1,2]. Here, we observed that STAg-pretreatment decreased the numbers of CD4^+^ T cells, CD11b^+^ monocytes, CD11b^+^CD11c^+^ DC and NK cells in the LP. The decreased numbers of CD4^+^ T cells in the LP could be contributed to the decreased immunopathology in the small intestine, since it was previously shown that this cell phonotype are involved in the intestinal lesions when C57BL/6 mice are infected with high doses of the parasite [1]. Additionally, we also observed that the STAg decreased the frequency of CD4^+^IFN-γ^+^ and CD4^+^IL-17^+^ T cells from the LP both cytokine previously shown to induce intestinal pathology in *T. gondii* infection [1,7,8]. Previous studies found DCs and macrophages in the LP [41], and under oral *T. gondii* infection Gr1^+^ granulocytes [42], inflammatory monocytes [Gr1^+^(Ly6C^+^, Ly6G^-^) F4/80 ^+^ CD11b^+^CD11c^-^] and CD11c^+^ cells [23] are found there. In *T. gondii* ileitis, IL-15-dependent gut NKp46 ^+^ NK1.1 ^+^ CD127^-^ LP NK cells produce the CCL3 that recruits inflammatory monocytes (Ly6C ^hi^F4/80 ^+^ Ly6G^-^CD11b^+^CD11c^-^) to the LP, where they enhance epithelial damage through their release of IL-1β, TNF-α and IL-6 [43]. NK cells were also identified as a major source of the IFN-γ that contributes to intestinal pathology and IL-17 in *T. gondii* infection [44,45]. In the current study, STAg-pretreatment decreased the number of these cells in the LP, which probably contributed to the decreased intestinal immunopathology.

We also found that STAg-pretreatment induced higher numbers of CD8^+^ IEL. CD8^+^ T lymphocytes in the intestinal epithelia produce substantial amounts of TGF-β and can inhibit IFN-γ production by CD4^+^ T cells in the LP [19]. Resistant CBA/J mice also depend on IEL-produced TGF-β to protect them against intestinal pathology from oral *T. gondii* infection [18], and these cells display cytotoxic activity, secrete cytokines and modulate epithelial cell death and regeneration [46]. To determine the roles of CD4^+^ and CD8^+^ T cells in the setting of STAg-pretreatment, we pretreated nude mice with STAg and infected with *T. gondii*. The STAg- and PBS-pretreated Swiss nude mice had prolonged survival rates, although both treatment groups died in the late stage of acute infection. These results demonstrate that following peroral inoculation, T cells contribute to early mortality but are necessary to control the parasite in later phases of infection. These data are in accordance with previous studies that demonstrated that BALB/c- or C57BL/6-background nude mice survived significantly longer than euthymic C57BL/6 mice when animals were infected with 100 ME-49 *T. gondii* cysts by oral route [1]. To further dissect the role of different T cell populations in the STAg-dependent protective effect, MHC class I- and II-deficient mice were pretreated with STAg and infected with *T. gondii*. STAg-pretreatment prolonged the survival of MHC class I^-/-^ and II^-/-^ mice, indicating, respectively, the presence of intestinal pathogenic CD8^+^ and CD4^+^ T cells during disease. Previous studies have shown a pathogenic role for CD4^+^ T cells in the intestine [1,2,19] and for a LP-derived CCR2 ^+^ CD4^+^ T cell population that emerged in the IEL compartment concomitant with lesion onset [47]. In addition, CD8αβ^+^ IEL from infected mice produce IFN-γ and display cytotoxic activity against infected enterocytes and macrophages in vitro [48], and CD8α^+^TCRαβ ^+^ CCR2^+^ IEL mediate inflammatory gut pathology during *T. gondii* infection [3]. We observed that there are more pathogenic CD8^+^ T cells than CD4^+^ T cells in oral *T. gondii* infection of C57BL/6 mice with high parasite load; MHC class I^-/-^ animals were more resistant to *T. gondii* when treated with either PBS or STAg than WT mice, although STAg-pretreatment confers higher resistance versus PBS. Despite previous studies demonstrated that the β-2 microglobulin-deficient mice develop intestinal pathology in the ileum as do C57BL/6 mice when infected with 100 *T. gondii* cysts [1]. Therefore, while CD8^+^ IEL have important roles in homeostasis and protection against infection, these cells display pathogenic activity under some inflammatory conditions. In the case of STAg-pretreatment, the recruitment of lesion-inducing CD8^+^ T cells into the intestinal epithelium may be replaced by protective IEL. In this context, despite our approach does not identify what these cell phenotypes are producing, we verified that the STAg-pretreatment reduce the frequency of CD3^+^CD8^+^IFN-γ^+^ and CD3^+^CD8^+^IL-17^+^ IELs.

With relation to NO, STAg-pretreated mice had decreased iNOS expression in the small intestine. There are CD11b^+^CD11c^+^Ly6C ^+^ MHC II^+^ inflammatory DC that produce high levels of iNOS [49]. Our results showed that STAg-pretreatment decreased the number of CD11b^+^CD11c^+^ DC in the LP and reduced iNOS expression. We also asked whether STAg-pretreatment would inhibit IL-17-expressing cells, as IL-17 is considered one of the cytokines responsible for the development of IBD [50]. We observed fewer ileum IL-17^+^ cells and decreased ileitis in STAg-pretreated mice compared to the PBS group. We postulate that IL-17^+^ cells, in addition to IFN-γ could be influencing gut tissue damage in PBS-pretreated mice.

In a different experimental model, and in accordance with our data, it was shown that STAg-pretreatment 4 days before allogeneic transplantation prolongs the survival of heart and skin allografts by reducing CD4^+^ and CD8^+^ cells in the allograft and serum IL-12, IL-2 and IL-17 and increasing serum IL-10 [51]. Finally, what components of STAg are inducing protection of intestinal pathology under *T. gondii* infection? By proteomic analysis of STAg it was isolated 11 proteins, a putative protein disulfide isomerase (PDI), heat shock protein 60 (Hsp60), a pyruvate kinase (PK), a putative glutamate dehydrogenase (GDH), a coronin, a heat shock protein 70 (Hsp70), a protein kinase C receptor 1 (RACK1), a malate dehydrogenase (MDH), a major surface antigen 1 (SAG1), an uridine phosphorylase (UPase) and a peroxiredoxin (Prx) [52]. Cyclophilin-18 constitutes on average of 1.7% of the total protein in STAg [21]. However, which STAg constituents modulate the inflammatory responses of the host and disease, as we demonstrated in the present investigation, is not known. Thus, studies are undertaken to determine what components of STAg are able to modulate the intestinal inflammation in oral *T. gondii* infection.

In conclusion, the data presented here suggest that the beneficial effects of STAg in controlling the vigorous proinflammatory immune response and tissue pathology are associated with an increase in protective CD8^+^ with smaller frequency of CD8^+^IFN-γ^+^ IEL in the small intestine along with a decrease in proinflammatory CD4^+^IFN-γ^+^ and CD8^+^IFN-γ^+^ T, NK, DC and monocytes in the LP, higher TGF-β expression and lower iNOS and IL-17 production.

## Supporting Information

Figure S1 **Mortality rate and parasitemia of BALB/c STAg-pretreated mice infected with *T. cruzi*.** BALB/c mice were STAg treated and 48h later infected by intraperitoneal route with 1000 trypomastigotes of *T. cruzi* (Y strain) and the mortality and parasitemia were accompanied. The STAg or PBS-treated animals presented the same pattern of susceptibility (χ^2^=0.03326; *p* = 0.8553; df = 1) (A). The parasitemia levels were evaluated in 5 µl of blood obtained from the tail vein (B). *p < 0.05 (Significantly different from values obtained from PBS-pretreated mice, Unpaired Student’s *t*-test).
(TIF)
