# Supplementary figures and images for: Toxoplasma gondii Soluble Tachyzoite Antigen Triggers Protective Mechanisms against Fatal Intestinal Pathology in Oral Infection of C57BL/6 Mice
Source: PLoS One. 2013 Sep 24;8(9):e75138. doi: 10.1371/journal.pone.0075138 (PMC3782460; doi:10.1371/journal.pone.0075138)

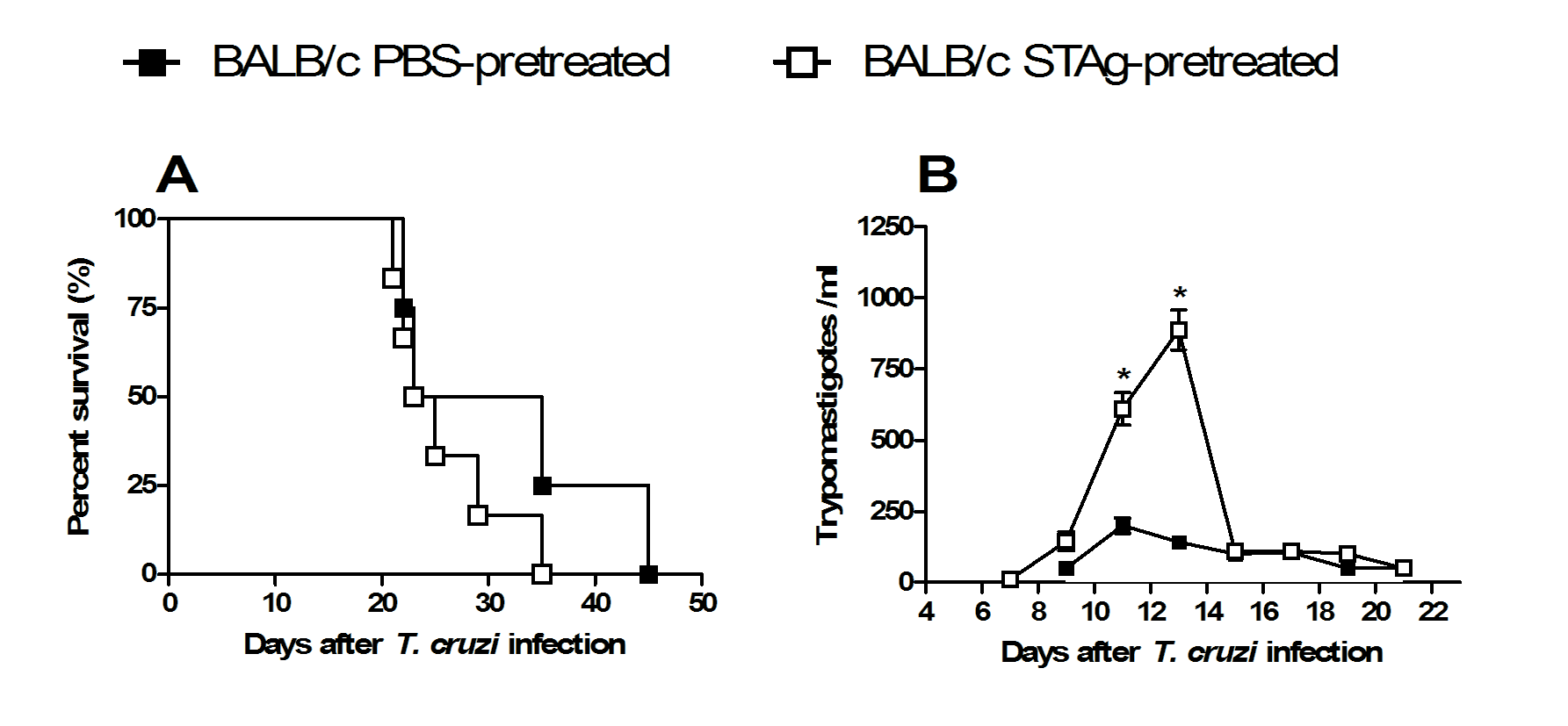

Supplement: Figure S1 — Mortality rate and parasitemia of BALB/c STAg-pretreated mice infected with T. cruzi. BALB/c mice were STAg treated and 48h later infected by intraperitoneal route with 1000 trypomastigotes of T. cruzi (Y strain) and the mortality and parasitemia were accompanied. The STAg or PBS-treated animals presented the same pattern of susceptibility (χ2=0.03326; p = 0.8553; df = 1) (A). The parasitemia levels were evaluated in 5 µl of blood obtained from the tail vein (B). *p < 0.05 (Significantly different from values obtained from PBS-pretreated mice, Unpaired Student’s t-test). (TIF) [file pone.0075138.s001.tif]
